# Deploying Metagenomics to Characterize Microbial Pathogens During Outbreak of Acute Febrile Illness Among Children in Tanzania

**DOI:** 10.3390/pathogens14060601

**Published:** 2025-06-19

**Authors:** Shabani Ramadhani Mziray, George Githinji, Zaydah R. de Laurent, Peter M. Mbelele, Khadija S. Mohammed, Boaz D. Wadugu, Brian S. Grundy, Scott K. Heysell, Stellah G. Mpagama, Jaffu O. Chilongola

**Affiliations:** 1 Department of Biochemistry and Molecular Biology, KCMC University, Moshi P.O. Box 2240, Tanzania; 2 Kibong’oto Infectious Diseases Hospital, Kilimanjaro P.O. Box 12, Tanzania; 3 KEMRI-Wellcome Trust Research Programme, Kilifi P.O. Box 230, Kenya; 4 Department of Biochemistry and Biotechnology, Pwani University, Kilifi P.O. Box 195-80108, Kenya; 5 Department of Microbiology and Immunology, Muhimbili University of Health and Allied Sciences, Dar Es Salaam P.O. Box 65001, Tanzania; 6 Kilimanjaro Clinical Research Institute, Moshi P.O. Box 2236, Tanzania; 7 Division of Infectious Diseases, University of Colorado, Aurora, CO 80045, USA; 8 Division of Infectious Diseases and International Health, University of Virginia, Charlottesville, VA 22903, USA

**Keywords:** metagenomic next-generation sequencing, mNGS, acute febrile illness

## Abstract

Outbreaks of infectious diseases contribute significantly to morbidity and mortality in resource-limited settings, yet the capacity to identify their etiology remains limited. We aimed to characterize microbes and antimicrobial resistance (AMR) genes in Tanzanian children affected by an acute febrile illness (AFI) outbreak using metagenomic next-generation sequencing (mNGS). A cross-sectional study was conducted on archived blood samples from children who presented with AFI between 2018 and 2019. Total nucleic acids were extracted from 200 µL of blood, and complementary DNA (cDNA), along with enriched pathogenic DNA, was sequenced using the Illumina MiSeq platform. mNGS data were analyzed using CZ-ID Illumina mNGS bioinformatics pipeline v7.0. Results were obtained from 25 participants (mean age: 11.6 years; SD ± 5), of whom 36% had a moderate to high-grade fever. The following five potential microbial causes of AFI were identified: *Escherichia coli* (n = 19), *Paraclostridium bifermentans* (n = 2), *Pegivirus C* (n = 2), *Shigella flexneri* (n = 1) and *Pseudomonas fluorescens* (n = 1), with *E. coli* being the most prevalent. Twelve AMR genes were detected, including *mdtC*, *acrF*, *mdtF*, and *emrB. E. coli* harbored most of the AMR genes previously associated with resistance to commonly used antibiotics. mNGS offers a promising complementary approach to conventional diagnostics for identifying pathogens and AMR profiles in vulnerable populations.

## 1. Introduction

Outbreaks caused by bacteria, viruses, protozoa, and fungi pose global health threats, with sub-Saharan Africa particularly vulnerable due to limited resources [1]. These outbreaks often present as syndromes, including viral hemorrhagic fevers (e.g., Ebola, Marburg), respiratory illnesses (e.g., influenza and COVID-19), and gastrointestinal infections attributable to various pathogens including *Vibrio cholerae*, *Salmonella* spp., *Shigella* spp., and *E. coli*. In settings like Tanzania, limited laboratory capacity hinders proper diagnosis, leading to empirical treatment of febrile illnesses as malaria [1,2]. However, not all fevers are due to malaria-causing pathogens like *Plasmodium falciparum*, underscoring the need for improved diagnostic capabilities to guide appropriate treatment and outbreak response.

Non-malarial acute febrile illnesses (AFIs) are frequent in resource limited settings like Tanzania and are associated with increased mortalities and morbidities [3,4]. Clinical presentations of non-malarial AFIs are similar to other febrile illnesses, including malaria, posing difficulties in diagnosis and interventions [5]. Etiological agents of non-malarial AFIs are often a highly diverse group of RNA viruses, particularly arboviruses, bacteria, fungi and protozoa [6,7]. These viruses are difficult to culture, making identifying and characterizing known and novel viral or bacterial components during outbreaks quite challenging. Some of the challenges in sub–Saharan Africa settings include the limited availability of diagnostics and high-throughput sequencing technologies for identifying both known and unknown pathogens. This is compounded by a shortage of human resources and infrastructural capacity, particularly in clinical and public health laboratories, which hampers the ability to identify the etiological agents responsible for various outbreaks [8]. Most of the laboratories deploy conventional blood culture as a gold standard for the identification of etiologies of blood stream infection due to bacteria or fungi. Yet, nearly half of the conventional blood cultures are reported as negative due to presence of non-cultivable pathogens in the samples [9].

Molecular tools, such as real-time and quantitative polymerase chain reaction (PCR) assays have revolutionized laboratory diagnostic methods. Technologies such as quantitative PCR-based TaqMan Array Card and Matrix-Assisted Laser Desorption/Ionization Time-of-Flight Mass Spectrometry have significantly reduced the turnaround time, evidence indicates they have limited sensitivity when used in whole blood and work better in culture positive specimens, respectively [10,11].

Sequencing platforms including the HiSeq, MiSeq and NextSeq 550 by Illumina (San Diego, CA, USA), Oxford Nanopore Technologies such as PromethION/MinION (Oxford, UK), and Ion Torrent by ThermoFisher Scientific (Waltham, MA, USA) have shown the potential to overcome the limitations of PCR-based technologies. These platforms employ various sequencing chemistries including paired-end sequencing, which enables the detection of minor pathogenic variants and mutations, such as deletions, inversions, and insertions; however, some of them are limited by the shorter length of the sequence reads produced [12].

Recent advances in sequencing and bioinformatics technologies have led to the development of high-throughput molecular technologies. These technologies offer improved accuracy and provide an opportunity to discover new genes or genomes of non-cultivable microorganisms in real time [13]. We aimed to evaluate a hypothesis free method to describe the etiological agents and their antimicrobial resistance genes associated with a fever outbreak that occurred in Tanzania in 2018 by performing unbiased metagenomic next-generation sequencing (mNGS) on blood samples from affected individuals.

## 2. Materials and Methods

### 2.1. Study Design and Setting

This cross-sectional study utilized archived blood samples from children enrolled in a malaria study conducted between April 2018 and June 2019 in the Korogwe District, Tanga Region, northeastern Tanzania. Korogwe is a high malaria-endemic area, with prevalence rates ranging from 14% to 37% [14], partly due to its high annual temperature (32 °C) and relative humidity (78.6%) [15]. According to the Global Campaign for Climate Action, Korogwe is located at longitude 38.4535628 and latitude −5.1609527, at an elevation of 305.48 m above sea level. The samples were archived at the Kilimanjaro Clinical Research Institute Biotechnology Laboratory in northern Tanzania, where all laboratory procedures were also performed.

### 2.2. Study Participants and Inclusion Criteria

Archived blood samples stored at −80 °C were examined. These samples were originally collected during an outbreak involving children presenting with an acute febrile illness (AFI), defined as an axillary temperature ≥ 38.3 °C. Samples had been preserved in an RNA stabilizer, TRIzol reagent (Molecular Research Center, Cincinnati, OH, USA) at a 1:3 ratio. Only samples that tested negative for malaria using the SD BIOLINE Malaria Ag P.f/Pan rapid diagnostic test were included. From a pool of 200 samples, 50 were randomly selected. Of these, 25 were included in the analysis, while the remaining 25 were excluded due to incomplete clinical or demographic data (age, sex, lactate, or hemoglobin levels).

### 2.3. Laboratory Procedures

#### 2.3.1. Total Nucleic Acid Extraction, cDNA Synthesis and Microbial Enrichment

Both RNA and DNA were co-extracted from 200 µL of each archived whole-blood sample using the Direct-zol DNA/RNA Miniprep Kit (Zymo Research Corp, Irvine, CA, USA), following the manufacturer’s instructions. Briefly, the extraction of nucleic acids was performed using spin-columns at a relative centrifugal force of 16,000× *g* for 1 min. Next, 50 μL of DNA/RNA was eluted using DNase/RNase-free water. Human ribosomal RNA was removed from the purified total RNA using the NEBNext rRNA Depletion Kit (New England BioLabs, Ipswich, MA, USA), as previously demonstrated by Butler and colleagues [16]. First-strand complementary DNA (cDNA) was synthesized using SuperScript™ IV Reverse Transcriptase (Thermo Fisher Scientific, Waltham, MA, USA) with random hexamers, following the manufacturer’s protocol. Second-strand cDNA synthesis was performed using 5U of Klenow Fragment (3′→5′ exo–) (New England BioLabs, Ipswich, MA, USA) based on an in-house optimized protocol. To enrich for potential etiologic pathogen DNA, the extracted DNA was processed using the NEBNext Microbiome DNA Enrichment Kit (New England BioLabs, Ipswich, MA, USA), as previously described by Feehery and colleagues [17]. DNA quantification was performed as needed using the Qubit Fluorometer v2 (Thermo Fisher Scientific, Waltham, MA, USA).

#### 2.3.2. Genomic Library Preparation and Sequencing

The genomic libraries were prepared using the Nextera XT DNA Preparation Kit (Illumina, San Diego, CA, USA). Briefly, 5 μL of input DNA was tagmented with an equal volume of amplicon tagment mix at 55 °C for 5 min, then held at 10 °C. The tagmented DNA was indexed using i5 and i7 primers with the Nextera PCR master mix. Indexing was performed using a limited-cycle PCR with the following conditions: 72 °C for 3 min; 95 °C for 30 s; 12 cycles of 95 °C for 10 s, 55 °C for 30 s, and 72 °C for 30 s; followed by 72 °C for 5 min, then held at 10 °C. Short DNA fragments in the PCR products were removed using 0.4× AMPure XP beads (Beckman Coulter, Brea, CA, USA). Libraries were normalized using library normalization beads (LNB1) and additives (LNA1) to ensure equal representation in the pooled sample. The pooled amplicon libraries were prepared by combining 5 μL of each normalized library, followed by denaturation at 96 °C for 2 min. Libraries were diluted to 8 pM, spiked with PhiX control, loaded into a 300-cycle reagent cartridge, and sequenced on an Illumina MiSeq (Illumina, San Diego, CA, USA).

### 2.4. Sequence Data Analysis

The FastQ sequence files generated were analyzed using a cloud-based Illumina mNGS pipeline v7.0 under the CZ-ID, formally IDseq [18]. All the pipeline user guides and scripts used for data analysis are open source and available in the GitHub repo at the following website: https://github.com/chanzuckerberg/czid-workflows, accessed on 10 May 2022. Briefly, the metagenomic pipeline had three major phases, as follows: (i) host filtering, (ii) assembly-based alignment, and (iii) taxonomic reporting. The human-derived raw sequence reads were filtered out using spliced transcripts alignment to a reference (STAR) by alignment to a HG38 reference database. The pipeline performed quality control checks by trimming the Illumina adapters and indices using trimmomatic [19]. The paired-read iterative contig extension (PRICE) computational package was used to remove the low-quality reads, duplicates, and low-complexity reads. Bowtie2 [20] was used to remove all the remaining human-derived sequence reads by aligning them against HG38 reference database. Taxa were assigned using an assembly-based alignment approach, which involved aligning the filtered reads against NCBI nucleotide and protein databases using a genomic short-read nucleotide alignment program (GSNAPL) [21] and RAPsearch2 [22], respectively. The short reads were de novo assembled using SPAdes [23] to generate contigs that would map the raw reads to identify their taxonomic belongings. The heatmap was produced at logarithmic scale to present the microbial relative abundance for each sample. The cut-off for determining the heatmap was set at 50 or more sequence reads per million (NT rPM ≥ 50), corresponding to taxon identified in the National Center for Biotechnology Information (NCBI) NT/NR database. Microorganisms with less than 50 NT rPM were considered not significant. Other filters used include nucleotide alignment length (NT L) ≥ 50, average percent identity of alignments to NCBI NT/NR (% ID) ≥ 95, E value (as of power of 10) less or equal to 0, and NT r (total reads) ≥ 5.

The resistance gene identifier (RGI) tool for AMR detection was implemented in the AMR pipeline v0.2.4-beta. Reads that passed quality filters and assembled contigs were compared to AMR reference sequences from the comprehensive antibiotic resistance database (CARD) using the RGI tool. The AMR genes with less than 10 rPM were regarded not significant and filtered out. Genomic data were partly visualized using Flourish tools, which are available at the following website: https://flourish.studio (accessed on 25 December 2024).

### 2.5. Statistical Analysis

Data were analyzed using IBM SPSS v23.0 (Chicago, IL, USA). Fisher’s Exact test was used to compare categorical variables at 2-sided level of significance. *p*-value of <5% was set as the cut-off for statistical significance. Based on the normality of the data, variable with continuous data were summarized by either the mean or the median with the corresponding measure of dispersion; standard deviation (SD) for normally distributed data and interquartile range (IQR) for non-normally distributed data.

## 3. Results

### 3.1. Demographic and Clinical Presentations

Out of the 50 selected participants’ blood samples, half were excluded and not processed using shotgun metagenomics next-generation sequencing (mNGS) due to missing demographic and clinical data. The remaining 25 (50%) were successfully sequenced using mNGS. The mean participants’ age was 11.6 years [SD, 5], with males comprising 13 (52%). Clinical measurements included mean body temperature of 39 °C [SD, 0.5], mean lactate of 4.7 mmol/L [SD, 2], and mean hemoglobin (Hb) of 12 g/dL [SD, 2]. Further characterization found that nine cases (36%) had moderate to high-grade fever (body temperature > 39 °C). In addition, nine (36%) participants had mild to moderate anemia, as described in Table 1.

### 3.2. Genomic Data Quality Metrics

**Figure 1:**
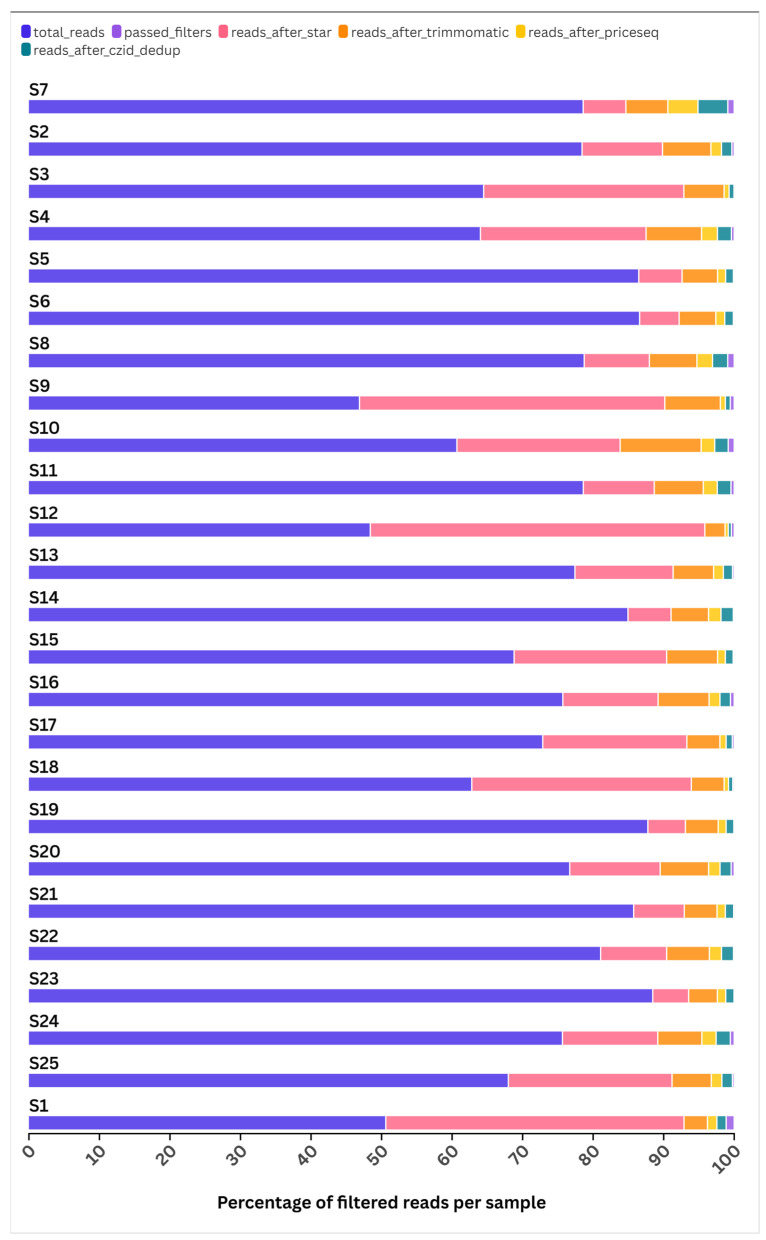
Chart showing percentage of reads passing filters for host cellular background per sample. The figure demonstrates significant proportions of reads that were filtered out using CZ-ID in silico filters. The chart was created with flourish.studio or equivalent available at https://flourish.studio (accessed on 25 December 2024).

The median number of sequences reads including host nucleic acids and potential etiologic pathogen per sample was 205,194 (IQR 56,045–458,288). Of these, the median number of reads that passed the host background filters was 626 (IQR 103–2206). A significant amount of host cellular background was eliminated using the CZ-ID pipeline tools, as illustrated in Figure 1. The filtering process allowed a small proportion (0.3% (626/205,194)) of filtered reads to advance to downstream in silico analysis within the pipeline. The median insert size was 85; however, it ranged from 4 to 2,800,766 (Table S1).

### 3.3. Detected Microorganisms

**Figure 2:**
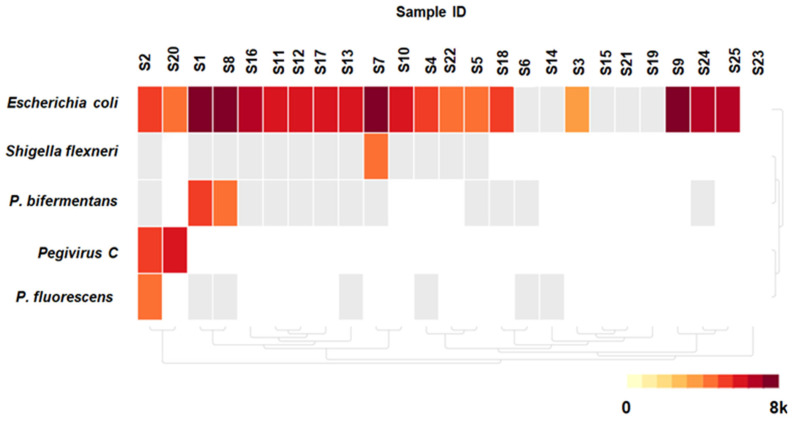
Heatmap presenting landscape of 5 microbial species across 25 samples in columns. Top purple row indicates sample collection location, which is Korogwe district, Tanga region in northern Tanzania. The amount of reads that correspond to taxa in NCBI NT/NR database are expressed in reads per million (rPM) on a colored logarithmic scale, where highest rPM is darker red. Boxes with gray color represent identified species that did not meet minimum threshold values. Map was created with CZ-ID tools.

Out of the 25 participants whose mNGS results were analyzed, potential microorganisms were detected in 19 (76%) participants (Figure 2). Eleven participants (57.9%) aged 1–12 years were identified with microorganisms, in contrast to their contemporaries lacking microbial identification. Although not statistically significant, nearly all the participants with microbial detection—18 (94.7%)—exhibited elevated serum lactic acid levels as compared to those without microbial detection, *p* = 0.430, as shown in Table 1. A total of five different microbial species were identified with a mean number of one microorganism detected per participant (range: 0–3).

Among the detected microbial species, four were classified as bacteria (80%) and one as a virus (20%). *Escherichia coli* was the most frequently identified species, found in 19 participants with a median of 814 [IQR 381–668] non-redundant reads per million per sample, corresponding to taxa in the NCBI NT/NR database. Co-detection with *E*. *coli* was noted in 5 out of 19 (26%), with the following combinations: sample ID 1, 2, 7, 8, and 20 detected (*E. coli* and *Paraclostridium bifermentans*), (*E*. *coli*, *Pegivirus C* and *Pseudomonas fluorescens*), (*E. coli* and *Shigella flexneri*), (*E. coli* and *Paraclostridium bifermentans*), and (*E. coli* and *Pegivirus C*), respectively. *Pegivirus C* was the only virus detected by mNGS and was observed in 2 out of 25 samples (Figure 2).

### 3.4. Reads Quality Metrics and Detection of Antimicrobial Resistance (AMR) Genes

**Figure 3:**
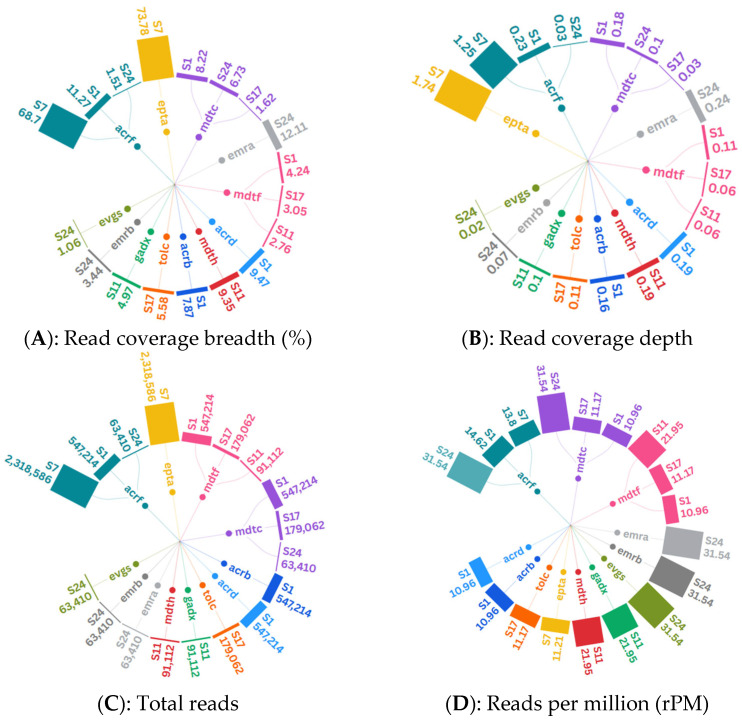
Radial tree charts portraying quality metrics of reads, of which 12 AMR genes were detected from five samples. Quality metrics are read coverage breadth, read coverage depth, total reads, and reads per million (rPM) described in panel (**A**–**D**)**,** respectively. Inner circles represent detected AMR genes in relation to metric per sample. Outermost bars represent sizes of metric per AMR gene identified in each sample. Chart was created with flourish.studio or equivalent available at https://flourish.studio (accessed on 24 December 2024).

The number of reads for AMR genes varied between 63,410 and 2,318,586 per sample. Sample ID 7 showed a significantly higher total number of reads (over 2.3 million), along with greater read coverage breadth (over 68%) and read coverage depth (over 1.25), compared to the AMR genes identified in other samples. In contrast, the AMR genes detected in sample ID 24 had higher number of reads per million (n ≥ 31) than those found in other samples (Figure 3A–D). A total of twelve AMR genes were identified in *E. coli* from five samples, namely *mdtC* (n = 3), *acrF* (n = 3), *mdtF* (n = 3), *evgS* (n = 1), *emrA* (n = 1), *emrB* (n = 1), *mdtH* (n = 1), *gadX* (n = 1), *eptA* (n = 1), *tolC* (n = 1), *acrB* (n = 1), and *acrD* (n = 1).

**Figure 4:**
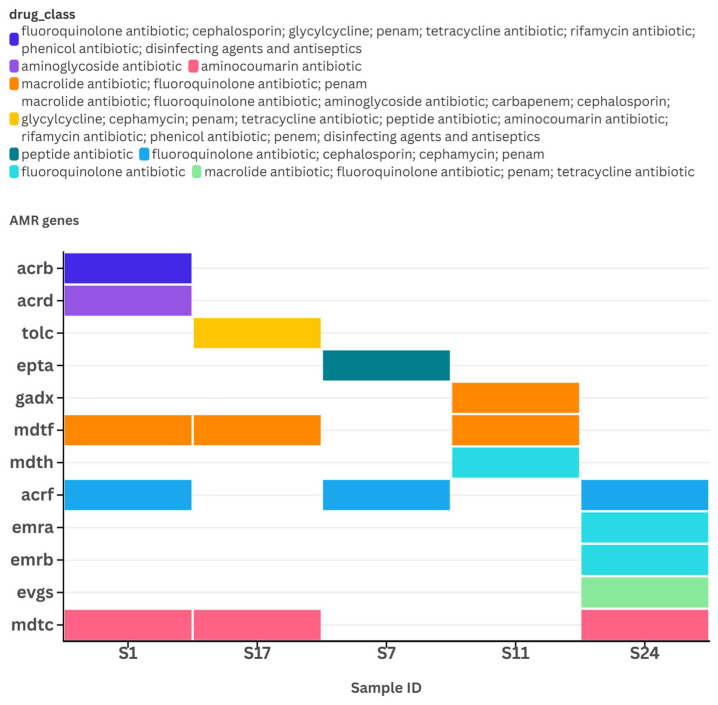
A categorical heatmap presenting antimicrobial resistance (AMR) genes of metagenomes isolated from five samples. Colored maps represent categories of drug class being affected by indicated AMR genes in each sample. Map was created with flourish.studio or equivalent available at https://flourish.studio (accessed on 24 December 2024).

Most of the identified genes were associated with a resistance–nodulation–cell division (RND) antibiotic efflux pump (Table S2). Samples with ID 1 and ID 24 contained bacteria harboring five different AMR genes, namely [*acrB*, *acrD*, *acrF*, *mdtC*, *mdtF*] in sample ID 1 and [*mdtC*, *evgS*, *emrB*, *emrA*, *acrF*] in sample ID 24, respectively. Each of these gene sets has the potential to confer resistance to five different drug classes. Notably, the genes *acrF, mdtC,* and *mdtF* were detected in three different samples (Figure 4). All identified AMR genes, except for *eptA*, affected different drug classes through an antibiotic efflux resistance mechanism. The *eptA* gene, which was only found in sample ID 7, was associated with an antibiotic target alteration resistance mechanism. Likewise, the *tolC* and *acrB* genes were reported to affect disinfecting agents and antiseptics (Table S2 and Figure 4).

## 4. Discussion

The application of shotgun metagenomic next-generation sequencing (mNGS), a technology known for its unbiased sequencing capabilities, revealed an increased relative abundance of *E. coli* in three-quarters of the children presenting with a non-malarial AFI. These findings suggest a potential cause of infectious AFI, such as transient translocation from another source, such as the gastrointestinal tract, or specimen contamination. However, if the mNGS findings of such a high frequency of *E. coli* bloodstream infections are accurate, they could explain a potential outbreak of non-malarial origin, possibly linked to a common hygiene-related source. This explanation was corroborated by the national multi-sectoral cholera prevention and control plan (2023–2027), which identified that, in 2019, the study setting was a high-risk hotspot for cholera due to poor water and latrine quality, as well as the consumption of contaminated water from the Pangani River [24]. Given that no further epidemiological information is known about the participants in the outbreak described, other correlations would be speculative.

Our study further identified *pegivirus C* in two participants. Similar to our findings, the detection of pegiviruses in people presenting with acute febrile illness has been previously documented in Tanzania, Gabon, and Brazil by using a similar sequencing technique, mNGS [25,26,27]. They are commonly co-detected with other viruses such as HIV, especially in severely immunosuppressed individuals [28]. A review of the literature shows that pegiviruses confer protective effects (better survival) in HIV co-infected individuals, and little is known about their potential to cause disease [29]. On the contrary, mNGS applied in this study did not detect HIV or other viruses in any of the 25 samples. They are ubiquitous blood-borne viruses and are often cleared by approximately three-quarters of infected immune-competent people [29].

Twelve AMR genes were detected from *E. coli*, namely *mdtC*, *acrF*, *mdtF*, *evgS*, *emrA*, *emrB*, *mdtH*, *gadX*, *eptA*, *tolC*, *acrB*, and *acrD*. With the exception of *eptA*, which was only found in sample ID 7 and associated with an antibiotic target alteration resistance mechanism, all other detected AMR genes were associated with multidrug resistance–nodulation–cell division (RND) superfamily exporters that affect different antibiotics—including fluoroquinolones, cephalosporins, and penams—by an energy-dependent antibiotic efflux resistance mechanism. In addition to the ability to confer multidrug resistance by efflux mechanisms, the encoded ArAB-TolC tripartite efflux pumps mediate the pathogenesis of *E. coli* [30]. Likewise, the ArAB-TolC tripartite efflux pumps increase the minimum inhibitory concentrations of many lipophilic/large drug molecules, such as cloxacillin, novobiocin, and erythromycin, making *E. coli* naturally resistant to those drugs. The ArAB-TolC tripartite efflux pump is located between the inner membrane, periplasm, and outer membrane of the bacterial cell and facilitates the complete pumping of drugs from inside the cell to the outside. However, despite the powerful nature of this tripartite pump, it cannot move aminoglycosides out; alternatively, the bacteria accomplishes this through the encoded AcrD pump, with the support of the AcrA and TolC pumps [30]. The AcrB and TolC proteins affect almost all kinds of antibacterials, including disinfecting agents and antiseptics, warranting the strengthening of infection and prevention control strategies, such as regular surveillance of AMR by culture and susceptibility testing of the bacteria isolated from various environments, including clinical and laboratory settings (e.g., benches, doorknobs, etc.).

The *eptA* gene encodes a phosphoethanolamine (pEtN) transferase that confers antibiotic resistance via target alteration, particularly against polymyxins such as colistin. The enzyme modifies the lipid A component of lipopolysaccharides (LPSs) in the outer membrane of Gram-negative bacteria by adding pEtN to the phosphate groups, thereby reducing the net negative surface charge. This modification diminishes the electrostatic interaction between colistin and the bacterial outer membrane, thereby reducing the antibiotic’s binding affinity and bactericidal activity. The expression of *eptA* is regulated by two-component systems, notably PmrAB (polymyxin resistance protein A and B) and PhoPQ (sensor protein PhoQ and a response regulator PhoP), which respond to environmental stimuli such as low magnesium levels; mutations in these systems can result in expression and persistent resistance. This mechanism has been documented in various pathogens including *Escherichia coli* and contributes to the growing challenge of treating infections with colistin, a last-resort antibiotic for treating drug-resistant Gram-negative bacteria [31].

The AMR genes were detected amid reports of an increased proportion of antimicrobial-resistant Gram-negative bacteria to commonly prescribed antibiotics, including third-generation cephalosporins, in Tanzania [32]. AMR genes are often expressed by common pathogens due to increased antimicrobial use [33]. AMR is usually associated with over-prescription; over-the-counter prescriptions; variations in adherence to treatment; and the consumption of animal products with antibiotic residues [34,35,36]. We advocate for the continued implementation of antimicrobial stewardship as one of the interventions towards reducing the growing burden of AMR in the country, in line with the National Action Plan on Antimicrobial Resistance 2023–2028 [37] and One Health Strategic Plan 2022–2027 in Tanzania [38], or their equivalents.

The mNGS technique has proven to be a useful tool in the surveillance of AMR in wastewater collected in the sewage networks of 60 countries worldwide, including Africa, Asia, South America, Europe, North America, and Oceania, as described by Hendriksen et al. in 2019 [39]. We also demonstrate its potential in the surveillance of AMR in clinical samples. Although the study used rigorous protocols to eliminate host RNA/DNA during wet and dry laboratory procedures, contamination might still have occurred in the results (sequence reads). This highlights the need for further optimization of both the wet and dry laboratory procedures for shotgun metagenomics to enhance the quality of data. In addition, metagenomic studies are very costly in terms of resources and time for optimization of procedures as compared to other molecular techniques, such as multiplex PCR. However, the standardization of protocols and adoption in the field will eventually lower the costs similar to current next-generation sequencing protocols.

## 5. Conclusions

*E. coli* is the most common microbial species detected in children presenting with a non-malarial AFI. This finding underscores the current understanding that not every fever is caused by malaria. In addition to current conventional diagnostic technologies for AFIs, mNGS is adaptable to support confirmation of etiological agents of AFI in an outbreak setting. Furthermore, the technology can be used to characterize microorganisms and corresponding AMR genes in samples collected during an AFI outbreak. We advocate for further optimization of the mNGS protocol, as well as its adaptation and uptake as a tool for characterizing etiological agents of known and unknown infectious diseases, especially in outbreak settings.

## Figures and Tables

**Table 1:** Demographic and clinical characteristics of participants disaggregated by mNGS microbial detection (N = 25).

Characteristic	Frequency	Microbial Detection by mNGS		
		Yes	No	
Age in years	n (%)	n (%)	n (%)	* *p*-value
1–12	13 (52)	11 (57.9)	2 (33.3)	0.378
≥13	12 (48)	8 (42.1)	4 (66.7)	
Mean age [SD]	11.6 [5]			
Sex				
Male	13 (52)	10 (52.6)	3 (50)	>0.99
Female	12 (48)	9 (47.4)	3 (50)	
Body temperature in °C				
38–39 (Mild fever)	16 (64)	13 (68.4)	3 (50)	0.335
>39–40 (Moderate fever)	7 (28)	4 (21.1)	3 (50)	
>40 (High grade fever)	2 (8)	2 (10.5)	0 (00)	
Mean temperature [SD]	39 [0.5]			
Lactic acid in mmol/L				
Normal (0.5–2.2)	2 (8)	1 (5.3)	1 (16.7)	0.430
Abnormal (>2.2)	23 (92)	18 (94.7)	5 (83.3)	
Mean lactate [SD]	4.7 [2]			
Hemoglobin (Hb) in g/dL				
>11.5	16 (64)	13 (68.4)	3 (50)	0.233
10–11.5	6 (24)	5 (26.3)	1 (16.7)	
8–<10	3 (12)	1 (5.3)	2 (33.3)	
Mean Hb [SD]	12 [2]			

* generated using Fisher’s Exact test. mNGS = shotgun metagenomics next-generation sequencing. SD = Standard Deviation. °C = Degrees Celsius.

## Data Availability

Metagenomic sequence data obtained in this study were deposited in the Sequence Reads Archive (SRA) of the NCBI database and are accessible through BioProject number PRJNA1013552, available at https://www.ncbi.nlm.nih.gov/sra/PRJNA1013552 (accessed on 6 September 2023), with accession number SRR25929716-40. Heatmaps and other features of the metagenomes analyzed are accessible at the CZ-ID database at https://czid.org (accessed on 10 May 2022), under the project named MetagenKorogwe. Clinical and demographic data are included in this manuscript as well as in the Supplementary Files.

## Supplementary Materials

The following supporting information can be downloaded at: https://www.mdpi.com/article/10.3390/pathogens14060601/s1, Table S1: Descriptions of sequence reads quality metrics; Table S2: Descriptions of antimicrobial resistance genes, affected drug classes and their resistance mechanisms.

## Abbreviations

The following abbreviations are used in this manuscript:

AFI	Acute febrile illness
AMR	Antimicrobial resistance
cDNA	Complementary DNA
CZ-ID	Chan–Zuckerberg mNGS bioinformatics pipeline
mNGS	Metagenomic next-generation sequencing
PCR	Polymerase chain reaction
rPM	Reads per million
