# Supplementary material for: Deploying Metagenomics to Characterize Microbial Pathogens During Outbreak of Acute Febrile Illness Among Children in Tanzania
Source: Pathogens. 2025 Jun 19;14(6):601. doi: 10.3390/pathogens14060601 (PMC12196098; doi:10.3390/pathogens14060601)
Supplement: Supplementary file 1 [file pathogens-14-00601-s001.zip › Table S1.pdf]

Table S1. Descriptions of sequence reads quality metrics. The file provides the outputs of CZ-ID bioinformatics pipeline tools that were used for host background filtration

sample_name	total_reads	passed_filters	passed_filters_percent	quality_control	compression_ratio	reads_after_star	reads_after_trimmomatic	reads_after_priceseq	reads_after_czid_dedup	insert_size_median	insert_size_min	insert_size_max	insert_size_mean	insert_size_standard_deviation
**S25**	4,810	18	0.374	6.456	1	1642	394	106	106	96	25	276110	112.426	82.2887
**S9**	6,056	72	1.189	1.645	1.02	5592	1018	92	90	69	14	582615	83.6318	61.4235
**S10**	17,200	240	1.395	8.293	1	6560	3256	544	544	71	19	672759	82.4581	51.984
**S15**	22,368	48	0.215	5.119	1	7032	2348	360	360	84	20	616406	98.2717	68.1657
**S21**	47,440	40	0.084	16.516	1	3972	2566	656	654	102	18	616317	118.085	88.0858
**S23**	48,680	14	0.029	23.269	1.01	2802	2242	652	648	112	20	553812	129.812	100.314
**S24**	63,410	462	0.729	14.954	1	11328	5240	1694	1690	83	19	635391	100.023	70.8129
**S11**	91,112	532	0.584	19.732	1.02	11656	8058	2300	2252	85	16	881657	104.863	75.6038
**S3**	101,934	44	0.043	2.47	1	44866	9032	1108	1106	101	13	581238	120.979	87.6425
**S22**	110,090	134	0.122	18.308	1.01	12716	8234	2328	2316	111	20	1189004	129.367	96.8986
**S20**	117,810	666	0.565	12.617	1.02	19704	10544	2486	2430	74	18	475075	90.9816	64.1864
**S17**	179,062	674	0.376	4.322	1.01	50210	11514	2170	2154	85	18	839314	102.794	69.4951
**S13**	205,194	632	0.308	9.881	1.05	36900	15220	3646	3462	84	15	828368	100.063	72.3862
**S18**	250,296	812	0.324	1.979	1.02	124076	18550	2456	2418	75	12	1355068	90.0322	60.6509

Table S1. Descriptions of sequence reads quality metrics. The file provides the outputs of CZ-ID bioinformatics pipeline tools that were used for host background filtration

**S19**	258,100	152	0.059	20.938	1.01	15656	13692	3278	3238	103	14	708044	121.079	89.0172
**S14**	344,284	508	0.148	28.819	1	24768	21552	7138	7110	106	19	1356901	124.126	92.2305
**S4**	369,928	2,276	0.615	9.585	1.13	135708	45440	13008	11486	70	11	1726607	90.0512	65.7371
**S8**	428,150	4,994	1.166	24.049	1.03	50230	36638	12080	11702	88	15	1095735	110.535	78.6997
**S12**	443,204	3,680	0.83	0.976	1.06	434346	26268	4238	3980	53	12	1782787	64.2637	42.3966
**S16**	473,372	3,308	0.699	11.415	1.03	84452	45192	9640	9360	78	15	880060	94.8861	67.7053
**S5**	487,374	626	0.128	18.527	1.02	34588	28386	6408	6274	90	19	769249	107.695	77.1057
**S2**	502,842	2,136	0.425	13.2	1.02	73152	43958	9656	9426	79	4	744060	99.668	71.4114
**S1**	547,214	12,338	2.255	3.154	1.01	457296	35962	14424	14244	62	10	1534030	73.7874	46.6166
**S6**	691,920	950	0.137	22.582	1.03	44780	41550	10112	9786	98	17	1770368	118.053	85.3537
**S7**	2,318,586	26,788	1.155	70.553	1.01	178504	175572	125940	124914	121	14	2800766	148.812	104.889
