# Supplementary material for: Deploying Metagenomics to Characterize Microbial Pathogens During Outbreak of Acute Febrile Illness Among Children in Tanzania
Source: Pathogens. 2025 Jun 19;14(6):601. doi: 10.3390/pathogens14060601 (PMC12196098; doi:10.3390/pathogens14060601)
Supplement: Supplementary file 1 [file pathogens-14-00601-s001.zip › Table S2.pdf]

Table S2. Descriptions of antimicrobial resistance genes, affected drug classes and their resistance mechanisms

Sample ID	AMR gene	AMR gene family	Affected drug class	Antimicrobial resistance mechanism	Bacterial species containing AMR gene
**S1**	acrb	resistance-nodulation-cell division (RND) antibiotic efflux pump	fluoroquinolone antibiotic; cephalosporin; glycylcycline; penam; tetracycline antibiotic; rifamycin antibiotic; phenicol antibiotic; disinfecting agents and antiseptics	antibiotic efflux	*Escherichia coli*
**S1**	acrd	resistance-nodulation-cell division (RND) antibiotic efflux pump	aminoglycoside antibiotic	antibiotic efflux	*Escherichia coli*
**S1**	mdtc	resistance-nodulation-cell division (RND) antibiotic efflux pump	aminocoumarin antibiotic	antibiotic efflux	*Escherichia coli*
**S1**	mdtf	resistance-nodulation-cell division (RND) antibiotic efflux pump	macrolide antibiotic; fluoroquinolone antibiotic; penam	antibiotic efflux	*Escherichia coli*
**S17**	mdtc	resistance-nodulation-cell division (RND) antibiotic efflux pump	aminocoumarin antibiotic	antibiotic efflux	*Escherichia coli*
**S17**	mdtf	resistance-nodulation-cell division (RND) antibiotic efflux pump	macrolide antibiotic; fluoroquinolone antibiotic; penam	antibiotic efflux	*Escherichia coli*
**S17**	tolc	ATP-binding cassette (ABC) antibiotic efflux pump; major facilitator superfamily (MFS) antibiotic efflux pump; resistance-nodulation-cell division (RND) antibiotic efflux pump	macrolide antibiotic; fluoroquinolone antibiotic; aminoglycoside antibiotic; carbapenem; cephalosporin; glycylcycline; cephamycin; penam; tetracycline antibiotic; peptide antibiotic; aminocoumarin antibiotic; rifamycin antibiotic; phenicol antibiotic; penem; disinfecting agents and antiseptics	antibiotic efflux	*Escherichia coli*

Table S2. Descriptions of antimicrobial resistance genes, affected drug classes and their resistance mechanisms

**S7**	epta	pmr phosphoethanolamine transferase	peptide antibiotic	antibiotic target alteration	*Escherichia coli*
**S7**	acrf	resistance-nodulation-cell division (RND) antibiotic efflux pump	fluoroquinolone antibiotic; cephalosporin; cephamycin; penam	antibiotic efflux	*Escherichia coli*
**S1**	acrf	resistance-nodulation-cell division (RND) antibiotic efflux pump	fluoroquinolone antibiotic; cephalosporin; cephamycin; penam	antibiotic efflux	*Escherichia coli*
**S11**	gadx	resistance-nodulation-cell division (RND) antibiotic efflux pump	macrolide antibiotic; fluoroquinolone antibiotic; penam	antibiotic efflux	*Escherichia coli*
**S11**	mdtf	resistance-nodulation-cell division (RND) antibiotic efflux pump	macrolide antibiotic; fluoroquinolone antibiotic; penam	antibiotic efflux	*Escherichia coli*
**S11**	mdth	major facilitator superfamily (MFS) antibiotic efflux pump	fluoroquinolone antibiotic	antibiotic efflux	*Escherichia coli*
**S24**	acrf	resistance-nodulation-cell division (RND) antibiotic efflux pump	fluoroquinolone antibiotic; cephalosporin; cephamycin; penam	antibiotic efflux	*Escherichia coli*
**S24**	emra	major facilitator superfamily (MFS) antibiotic efflux pump	fluoroquinolone antibiotic	antibiotic efflux	*Escherichia coli*
**S24**	emrb	major facilitator superfamily (MFS) antibiotic efflux pump	fluoroquinolone antibiotic	antibiotic efflux	*Escherichia coli*
**S24**	evgs	major facilitator superfamily (MFS) antibiotic efflux pump; resistance-nodulation-cell division (RND) antibiotic efflux pump	macrolide antibiotic; fluoroquinolone antibiotic; penam; tetracycline antibiotic	antibiotic efflux	*Escherichia coli*
**S24**	mdtc	resistance-nodulation-cell division (RND) antibiotic efflux pump	aminocoumarin antibiotic	antibiotic efflux	*Escherichia coli*
